# A Review of Non-cultured Epidermal Cellular Grafting in Vitiligo

**DOI:** 10.4103/0974-2077.79181

**Published:** 2011

**Authors:** Nanja van Geel, Boon Kee Goh, Elien Wallaeys, Stefanie De Keyser, Jo Lambert

**Affiliations:** *Department of Dermatology, Ghent University Hospital, De Pintelaan, Ghent, Belgium*; 1 *Pigment Clinic, National Skin Center, Mandalay Road, Singapore*

**Keywords:** Melanocyte transplantation, cellular grafting, surgical treatment, vitiligo

## Abstract

Non-cultured epidermal cellular grafting is an innovative surgical technique that can be used for the treatment of stabilized leucoderma, including vitiligo. Many reports have been published since its introduction in 1992, including several modifications and simplification of the original technique. This systematic review gives an overview of the literature.

## INTRODUCTION

Many treatment modalities are currently used for vitiligo, such as psoralen plus ultraviolet A (PUVA), narrowband ultraviolet B (NB-UVB), excimer lasers, topical steroids, topical immunomodulators, and calcipotriol. In patients with stable leucoderma (e.g., segmental vitiligo), surgical methods can be alternative therapeutic options. These surgical techniques are based on a common principle: to transplant autologous melanocytes from a normal pigmented donor skin to depigmented area. Many surgical techniques for repigmenting vitiligo have been devised over the years and can be broadly divided into tissue and cellular grafting. Tissue grafts include full-thickness punch grafts, thin dermoepidermal grafts, and suction epidermal grafting. With these tissue grafts, only a limited surface area can be treated per treatment session. Cellular grafts include cultured pure melanocytes suspension and non-cultured epidermal cellular suspensions (mixture of melanocytes and keratinocytes). These epidermal cells can also be co-cultured to epithelial sheet grafts. The major advantage of these suspension and culturing techniques is that, they permit treatment of affected skin manifold larger than the donor area. However, culturing techniques are time consuming, expensive due to the culturing time of several weeks, and require highly trained personnel and well-equipped tissue laboratories. Furthermore, the use of specific growth factors and additives in the culture medium (e.g., 12-O-tetradecanoyl-phorbol 13-acetate/TPA), pose safety concerns. These limitations were overcome with the introduction of the non-cultured cellular grafting techniques in 1992 by Gauthier and Surleve-Bazeille.[1] With this technique a cellular suspension is used without first expanding the cells in culture. Larger areas (8-to10-fold size of donor skin), can be treated and the procedure can be completed in several hours in an outpatient basis. In a double-blind placebo-controlled study, published in 2004, we demonstrated that repigmentation was primarily induced by the transplanted melanocytes and not by the skin abrasion.[2] This has also been reported earlier by Olsson *et al*.[3–4]

Since the description of the original procedure in 1992, many reports followed, including several modifications and further simplifications. The purpose of this review is to give an overview of the literature with respect to treatment outcome and developments in the technical procedure of non-cultured cellular grafting in vitiligo.

## METHODS FOR DATA COLLECTION

The computerized bibliographical databases Pubmed (U.S. National Library of Medicine; National Institutes of Health) was screened for clinical trials from January 1960 to July 2010. The main keywords used were “vitiligo, grafting, melanocyte grafting, surgery, melanocyte transplantation, non-cultured epidermal grafting.” Other data sources were the reference lists from articles retrieved. Only the clinical trials on non-cultured epidermal cellular grafting (using trypsin) for vitiligo (segmental, generalized, mixed) and in addition, other types of leucoderma (halo nevi, nevus depigmentosus, chemical leucoderma, piebaldism) were included. Single case reports were not included. All publications were classified by level of evidence (CBO/EBRO guidelines, Utrecht, The Netherlands).

### Classification level of evidence (CBO/EBRO guidelines, Utrecht, The Netherland)

**Table:** 

A1	Meta-analysis containing at least some trials of level A2 and of which the results of individual trials are consistent
A2	Randomized comparative clinical trials of good quality (randomized double-blind controlled trials) of sufficient size and consistency
B	Randomized clinical trials of moderate (weak) quality or insufficient size or other comparative trials (non-randomized, cohort studies, patient–control studies)
C	Non-comparative trials
D	Expert opinion

### Results of literature review

In total, 21 studies were obtained using the database [Table 1]. Most of them (16) were publications of level of evidence C, 3 of evidence level B, 2 of evidence level A2, and none of evidence level A1.

**Table 1:** Overview of literature concerning non-cultured cellular grafting in leucoderma

Author	Evidence level	Follow-up	N	Treatment indication	0%–24%	Repigmentation achieved in x%		95%–100%	Repigmentation >75%	Different scores of repigmentation (achieved in 25%–64% 65%–94% x% of patients) Comments
						25%–64%	65%–94%			
Gauthier, 1992[1]	C	1–3 months	12	FV, ND, SV	33	0	42	25	50	
Olsson, 1998[6]	C	6–12 months	26	CL, GV, HN, ND, P, SV	8	15	23	54	73	
van Geel, 2001[7]	C	6–20 months	4	GV, SV	0	0	100	0	100	
Olsson, 2002[15]	C	1–7 years	52*	FV, GV, HN, P, SV	N.R	N.R.	19**	21**	N.R.	0–19 repigmentation:39** 20–64 repigmentation: 21**
Issa, 2003[18]	B	3 months	11	GV	N.R.	N.R.	N.R.	N.R.	N.R.	“Significant difference compared to initial area”
Mulekar, 2003[19]	C	1 year	184	FV, GV, SV	GV: 23 SV: 4 FV: 26	GV: 9 SV: 0 FV: 0	GV: 8 SV: 12 FV: 0	GV: 53 SV: 84 FV: 69	N.R.	
van Geel, 2004[2]	A2	3–12 months	26	GV	57	0	15	27	42	
Mulekar, 2004[17]	C	1–5 years	64	FV, SV	SV: 10 FV: 20	SV:0 FV:7	SV:6 FV:0	SV: 84 FV: 73	N.R.	
Mulekar, 2005[20]	C	1–6 years	142	GV	24	9	11	56	N.R.	
Pandya, 2005[21]	C	1–6 months	23	SV, GV	11	11	N.R.	N.R.	N.R.	65–90 repigm.: 18,5 >90 repigm.: 52,2
Xu, 2005[22]	C	6–18 months	24	FV, GV, SV	N.R.	N.R.	N.R.	N.R.	N.R.	62–93 repigm.: (after 1 treatment): 58 85–97 repigm.: (after 2 treatments): 38
Tegta, 2006[23]	B	3 months	20	FV, GV, SV	Group A: 20# Group B: 70##	N.R.	N.R.	N.R.	Group A: 50 Group B: 0	26–50 repigm.: Group A: 10; Group B: 20 51–75 repigm.: Group A: 20; Group B: 10
van Geel, 2006[24]	C	3–12 months	39	FV, GV, SV	10	21	43	26	59	
Mulekar, 2008[11]	B	4 months	5	GV, SV	ReCell: 20 Conv Tx:20	ReCell: 20 Conv Tx: 20	ReCell: 20 Conv Tx: 0	ReCell: 40 Conv Tx: 60	ReCell: 40 Conv Tx: 60	
Mulekar, 2009[13]	C	6–12 months	49	SV, GV	18**	10**	32**	40**	N.R.	Treatments on ‘difficult sites’
Back, 2009[14]	A2	1 year	13	GV §	N.R.	N.R.	N.R.	N.R.	N.R.	No repigm.: 84 Minimal repigm.: 8 Normal to hyperpigm.: 8
Cervelli, 2009[25]	C	2 years	15	FV, GV, SV	0	N.R.	N.R.	N.R.	80	Recell 25–50 repigm.: 20
Mulekar, 2010[16]	C	4 years	25	FV, SV	FV: 8 SV: 15	FV: 8 SV: 8	FV: 8 SV: 15	FV: 75 SV: 62	FV: 83 SV: 80	Only children included
Goh, 2010[10]	C	6 months	5	FV, P, SV	0	0	100	0	60	6-well plate technique
El-Zawahry, 2010[26]	C	6–17 months	22	FV, SV, GV	27	32	18	23	27	
Van Geel, 2010[5]	C	1–62 months	82	GV, SV, HN, P, ND, MV	12	12	33	43	70.7	

N = number of patients evaluated; GV, generalized vitiligo; SV, segmental vitiligo; FV, focal vitiligo; MV, mixed vitiligo; UV, unilateral vitiligo; HN, halo nevi; ND, nevus depigmentosus; P, piebaldism; CL, chemical leucoderma; Conv Tx, conventional transplantation; Repigm, repigmentation

* Number of locations treated

** percentage repigmentation per treated locations; NR, not reported

# Threefold of the donor site used

## Fivefold of the donor site used

§ In 69% active disease

### General information with respect to non-cultured epidermal cellular grafting

It is generally agreed, according to the collected literature that, several prerequisites are necessary for vitiligo patients to be eligible for cellular transplantation. Proper selection of patients is an essential requirement for successful repigmentation. The most important selection criterion is the stability of the disease. According to the majority of authors, vitiligo can be classified as being “stable,” when progression of old lesions and/or development of new lesions are/is absent in the past one year. As segmental vitiligo stabilizes spontaneously, in general, within the first year of onset, this remains the best surgical treatment indication.[5] Only a minority of patients with generalized vitiligo are suitable for a surgical intervention, as this type of vitiligo in general extends over time. Before treatment is initiated in patients with generalized vitiligo, it has to be pointed out that the intervention does not alter the underlying patho-aetiology and, despite the treatment, the natural course of the disease remains the same. Furthermore, the patients should not have a history of a hypertrophic scars and preferably have no signs of a Koebner phenomenon because the latter may negatively influence surgical treatment results.[2]

### Technical procedure of non-cultured cellular grafting

In the original description of the procedure, a donor sample was obtained from the scalp by superficial shaving using a dermatome with a razor blade and then treated with trypsin 0.25% for 18 h for dermo-epidermal separation.[1] Subsequently, epidermal cells were extracted and a cellular suspension was prepared. This suspension was inoculated into blisters raised with liquid nitrogen at the recipient area. The roof of an intact blister served as a natural dressing that held the transplanted cells in place.

In 1998, Olsson and colleagues described a comparable technique, but the donor skin was taken from the gluteal region using a Goulian biopsy knife. The time for trypsinization was reduced to 50 min and the cellular suspension was directly applied onto a dermabraded vitiligo lesion. This made it possible to perform the whole procedure on the same day.[6] A crucial limiting factor of this grafting technique, at least in our experience, was the fixation of the liquid suspension at the recipient area. To overcome this problem, we introduced hyaluronic acid as a biodegradable cell carrier to increase the viscosity of the suspension.[7]

Advances in the procedure were also made in the preparation of the recipient area. De-epithelization of the graft recipient area bed can be achieved by cryotherapy, induction of suction blisters or dermabrasion using a high-speed dermabrader. However, for more delicate or critical anatomic areas (e.g., eyelid) and for irregular or spotted lesions, laser abrasion [e.g., erbium:YAG or carbon dioxide (CO_2_) laser] can be a better alternative, as it offers excellent precision over margin and depth control. A histological study, comparing pulsed CO_2_ laser abrasion with conventional dermabrasion (standard Hall dermabrader burr) demonstrated that tissue destruction of the dermis was similar and that short-pulsed CO_2_ laser abrasion did not cause significant thermal necrosis on the surface of the papillary dermis to interfere with a satisfactory graft take.[8] However, for suction blister grafting, better results were achieved on recipient sites previously prepared with the suction blister technique compared to CO_2_ laser abrasion in a single case report.[9] But, the preferred technique for recipient area depends upon the choice of the surgeon.

**Figure 1:**
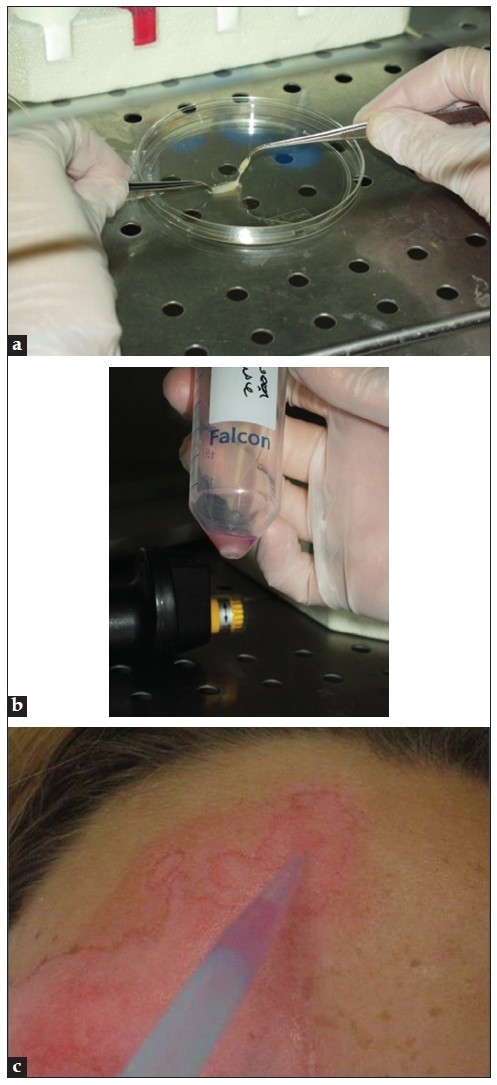
Preparation of non-cultured epidermal cellular suspension at Ghent University Hospital, Belgium; (a) Dermo-epidermal separation after trypsinization; (b) obtained cell pellet; and (c) cell suspension application on recipient area

There is ongoing research to simplify the procedure of non-cultured cellular grafting, and modifications with respect to safety of the procedure should further be taken into account [Figure 1a–c]. At least in our experience, the use of special culture media can be replaced by phosphate-buffered saline (PBS) or saline solution without xenobiotics (e.g., bovine serum or pituitary extract or other foreign proteins). Bovine serum, as a neutralizing agent for trypsin, can be substituted by soybean-derived trypsin inhibitor or autologous human serum drawn from the patient. To further simplify the laboratory procedure, one could also use the simplified cellular grafting technique described by Goh *et al*.[10] This modification reduces cell preparation time, amount of reagents needed and costs, and obviates the need of a laboratory for extraction of cells. With this technique, the extraction of epidermal cells from the donor skin is performed by using an inexpensive 6-well plate, a microfilter, and 3 reagents: trypsin, soybean trypsin inhibitor, and PBS. A comparable commercial kit (ReCell kit) is also available for the extraction of epidermal cells without the need of a laboratory.[11] However, this kit is expensive, which limits its use for routine use. One comparison study (n=5) between the commercial kit and the conventional method of cell suspension preparation, suggests that repigmentation was comparable for both the techniques used.[11] However, comparable studies on larger study populations are still missing.

The most simplified method would be to replace the enzymatic preparation of a cellular suspension by mechanical separation of epidermal cells. This is reported by Kachhawa, in which he uses epidermal scrapings (till the level of the papillary dermis) to apply directly onto the dermabraded recipient area.[12] However, questions regarding the homogeneity of the repigmentation, possible multiplication factor from donor skin to treatment area and treatment outcome on larger surface areas remain to be investigated.

### Treatment results

**Figure 2:**
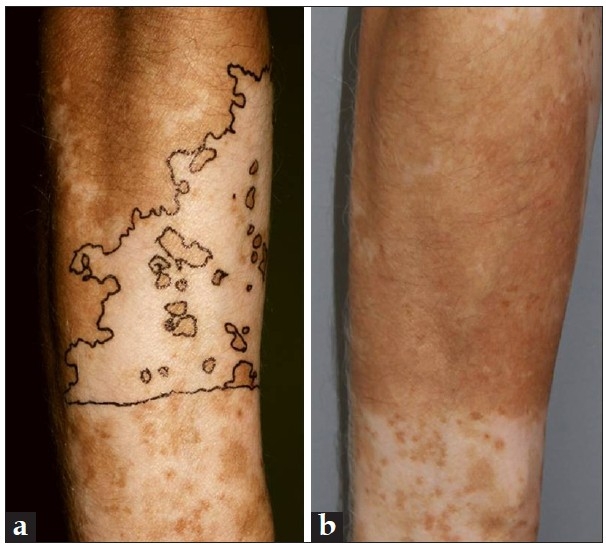
(a) A patient with segmental vitiligo on the right arm; (b) Repigmentation after non-cultured cellular grafting

With increasing experience, cumulative data on the outcome of non-cultured cellular grafting in the treatment of vitiligo and other types of leucoderma began to emerge. A summary of the literature, including treatment outcomes of different types of leucoderma, is show in Table 1. The repigmentation rates vary widely within the different publications (>75% repigmentation in 27-100% of patients). However, this might be explained by the fact that many differences exist among the studies, for example, different follow-up periods (1 month to 7 years), different characteristics of the included study populations (disease activity, treatment indication, treatment localization, Koebner phenomenon), different evaluation methods to assess repigmentation used, as well as variations in preparation of the cell suspension and post-operative treatment. This heterogeneity makes comparison among the studies difficult. However, several conclusions can be drawn from the available literature. Best results were in general, achieved in patients with completely stable leucoderma (segmental vitiligo [Figure 2a–b] and piebaldism). Anatomic sites that showed good repigmentation response were face/neck and trunk, while more resistant sites include the fingers and lips. Repigmentation of these “lip-tip” areas was more successfully achieved with mini-grafting, suggesting that the dermal component of the graft may influence treatment outcome. Mulekar *et al*, reported, more recently, good results with epidermal cellular grafting on “more difficult-to-treat sites” too (fingers, toes, elbows, …), but they mentioned that multiple sessions were often necessary.[13]

Repigmentation rates for segmental vitiligo is in general consistently high, regardless of cell preparation or dermabrasion technique used, while results in generalized vitiligo is variable and often more disappointing. Only one study included mixed vitiligo patients, but achieved inferior results in this group of patients.[5] The importance of disease stability has been demonstrated by many authors and most likely explains the poor results as achieved by Back *et al*.[14] In their randomized controlled trial, 69% of the 13 included patients, had an active generalized vitiligo. Furthermore, Koebner phenomenon was observed in 77% of their patients, which is known as an essential negatively influencing factor for surgical treatment outcome in vitiligo.[2,15]

### Adverse effects

Reported adverse effects on recipient and donor areas are in general minimal or absent according to the available literature. However, a frequently reported limitation is the presence of colour mismatch between the treated area and the surrounding skin. We described in a long-term follow-up study on non-cultured cellular grafting, the presence of some colour mismatch at the recipient area (hyper- and hypo-pigmentation) in 80.4% of 54 evaluated patients.[5] But, this colour mismatch was not disturbing according to the majority of these patients. At the donor site minor textural skin changes were observed in this study in 65% of the patients.[5] The majority of these patients accepted this side effect without any problem, as this was almost not visible by clinical inspection. Hyperpigmentation at the donor site, has been reported too by several authors.[16]

### Long-term results

Long-term follow-up studies on non-cultured epidermal cell transplantation suggest that it is effective and safe, particularly for repigmenting stable and localized types of leucoderma. Olsson and Juhlin reported in their follow-up study (1-7 years), results of 52 treated locations.[15] For segmental vitiligo and piebaldism, all patients achieved and retained 95%-100% repigmentation. However, for generalized vitiligo, they achieved only an average repigmentation of 49% in long-term evaluation. Similar results were reported by Mulekar (follow-up period of 1-5 years).[17] They reported good results in segmental and focal vitiligo. Repigmentation was retained until the end of the respective follow-up period. In our long-term follow-up study (1-7.7 years), repigmentation was retained in 93.3% of the grafted areas.[5] Loss of pigmentation was only observed by generalized vitiligo patients and not in segmental vitiligo, piebaldism, and halo nevi and nevus depigmentosus.

## CONCLUSION

A review of the available literature was performed to assess the effectiveness and developments in the technical procedure of non-cultured cellular grafting. Non-cultured epidermal cell transplantation provides the possibility to repigment vitiliginous skin manifold larger than the donor skin and can be completed in several hours on an outpatient basis. The selection of patients plays a significant role in achieving a successful repigmentation. Results are excellent in segmental vitiligo, halo nevi, piebaldism, and less in generalized vitiligo. Differences in these selection criteria may explain the variation in treatment outcome. Although this technique offers many advantages compared with the other surgical techniques, it requires highly trained personnel and well-equipped facilities. However, several advances have been made in recent years with respect to simplification of the laboratory procedure and safety.
